# Special Issue “The Genetic Changes Induced by Engineered Manufactured Nanomaterials (EMNs)”

**DOI:** 10.3390/nano12132233

**Published:** 2022-06-29

**Authors:** Marta Marmiroli

**Affiliations:** Department of Chemistry, Life Science, and Environmental Sustainability, University of Parma, Parco Area delle Scienze 33/A, 43124 Parma, Italy; marta.marmiroli@unipr.it

The possibility that engineered manufactured nanomaterials (ENMs) can be harmful to the genetic materials of living individuals has been highlighted in several experiments, but it is still controversial. In fact, there is also evidence that nanoparticles are not genotoxic and do not interfere with the genetic material of organisms. It is of extreme importance to establish which nanomaterials have the potential to exert harmful effects on DNA in different types of living organisms, from simple prokaryotes to complex eukaryotes, starting from model organisms. The aims and scopes of this Special Issue are to (1) highlight the research applications that identify which ENMs are genotoxic, and which are the more susceptible organisms or cell lines, and (2) to pinpoint reliable methods to establish the genotoxicity of ENMs [1].

Because of their large-scale manufacture and widespread application, several studies related to the toxicological assessment of nanomaterials (NMs) have been conducted over the past decade. Notwithstanding the extensive research on the cytotoxicity of NMs, their possible genotoxicity is of concern due to their increased utilization [2]. As explained in one of the reviews included in this Special Issue, the number and quantity of nanomaterials is ever increasing and affecting the environment where humans, bacteria, and plants live, and their genome come in contact with nanomaterials [3]. Although the topic of genotoxicity induced by nanomaterials is important, we had only five contributions for this Special Issue: [3,4,5,6,7,8].

Marmiroli et al., 2022 [3], contributed a minireview on the methods used to analyze genotoxicity in plants. Many plant species have the capability of being used as systems for genetic assays. Different mechanisms can be utilized according to the different ENM physico-chemical properties, specifically the following: (i) ENMs are able to pass through the cellular membrane lipid bilayer; (ii) endocytosis processes, the Trojan horse mechanism and biotransformation processes drive the accumulation of ENMs in plant cells; (iii) the utilization of membrane transporters mediating the translocation into the plant cell. These phenomena cause the interaction of ENM with DNA and chromatin and standard methods to measure the damages that can be caused are revised [3].

Lizzi et al., 2021 [4], studied the effects of multiple applications of CeO_2_ oxide nanoparticles on a wild plant, *Silene flos-cuculi*, instead of a classical crop or model plants. They measured the quantity of nanoparticles in the plants utilising a spICP-MS (Single Particle ICP-MS), and other parameters related to the plant biomass. They found that the CeO_2_ nanoparticles translocated from roots to shoots and had adverse effect on the plant health, which indicates possible damage to the organellar DNA. However, the nanoparticles genotoxicity was not measured directly.

Ma et al., 2021 [5], analyzed the consequences of the application of Graphitic carbon nitride nanosheets (C_3_N_4_) on rice plants (*Oryza sativa*) grown on soils contaminated with Cd and As. They found that not only did the nanomaterials increase the yield of the plants, but they abated the genotoxicity caused by Cd. Cd genotoxicity was studied through the application of a random amplified polymorphic DNA (RAPD) analysis. The RAPD primer used in this assay was OPC20 (ACT TCG CCA C). They also analysed the expression of many transporters of As and Cd under the effect of C_3_N_4_, finding them mostly downregulated thanks to the presence of the nanomaterial. Therefore, C_3_N_4_ may be a promising material that is sustainable for safe nano-enabled strategies of reducing heavy metal accumulation in key food crops grown in contaminated soils.

Gallo et al., 2021 [6], conducted a proteomic study of two *Arabidopsis thaliana* (L.) Heynh mutants resistant to lethal amounts of CdS Quantum Dot (QD) for the wild type. In fact, in a previous work, two independent *Arabidopsis thaliana* Ac/Ds transposon insertional mutant lines, atnp01 and atnp02, were identified. The tolerance response was completely characterized [7]. In this work, a comparative analysis was performed on protein extracts from plantlets of the two mutants and of wt, each treated with a sublethal concentration of CdS QDs. Two Dimension-PAGE was used to conduct a comparative protein analysis; proteins were characterized by MALDI-TOF/TOF. Ninety eight of the proteins identified showed significant changes in their relative abundance between control and CdS QD-treated plantlets. The two mutants showed a different response to the treatment regarding the type and quantity of up- and downregulated proteins. This difference became more striking when compared to wt. The proteins were analyzed through GO and MapMan to identify functions and pathways. A network analysis of the proteins differentially expressed in the two mutants showed that several of the proteins encoded by putative genes contained transposons, which were responsible for the regulation of some proteins identified in this study. These proteins included complex 3 (Elo3) which is involved in transcriptional elongation; nifu-like protein 3 (Nfu3) which is involved in chloroplast assembly; protein phosphatase 2C (PP2C) which mediates abiotic stress response; magnesium-chelate subunit-2 (Chli2) which is involved in chlorophyll biosynthesis; and other relevant proteins. The change in the protein regulation due to CdS QDs may be due to an interference of the QDs with the DNA and the transcription.

Wu and colleagues, 2021 [8], documented the possible genotoxicity of graphene in all its form to human cells. The graphene nanomaterials family (GFNs) includes graphene, graphene oxide (GO), reduced graphene oxide (rGO), and graphene quantum dots (GQDs). They have a wide range of potential applications, creating the possibility of their release into the environment which implicates exposure to humans and other organisms. However, the genotoxicity of GFNs to DNA remains largely unknown. In their review, the authors studied the interactions between DNA and GFNs and pinpointed the mechanisms of genotoxicity caused by GFNs. In general, genotoxicity can be classified into direct genotoxicity and indirect genotoxicity. The two types of genotoxicity (e.g., direct physical nucleus and DNA damage; and indirect physical destruction, oxidative stress, epigenetic toxicity, and DNA replication) of GFNs were also explored in the paper. Additionally, the influencing factors of the nanoparticles and of the type of experiment (e.g., physicochemical properties, exposure time and dose, the genotoxicity of GFNs) were taken into consideration. The authors conclude that considering the key role of genotoxicity in GFNs’ exposure risk assessment, future research is warranted.
